# A topic among others—examining the attention dynamics of the COVID-19 pandemic through interviews with Finnish journalists

**DOI:** 10.1177/14648849221138431

**Published:** 2022-11-02

**Authors:** Timo Harjuniemi

**Affiliations:** Demos Research Institute & University of Helsinki, Helsinki, Finland

**Keywords:** Issue attention, COVID-19, news media, pandemics, attention cycle

## Abstract

Media research has shown that media attention to pandemics is largely driven by rising case numbers, policy responses and scientific breakthroughs. However, less is known about the issue attention dynamics that feed a decline in the media attention to pandemics. By building on the literature on issue attention and on 18 semi-structured interviews with Finnish journalists, this article explores the issue attention dynamics of the COVID-19 pandemic. The article identifies three factors that lead to a decrease in the attention that a pandemic receives. First, issue fatigue diminishes the amount of attention while issue competition replaces the pandemic with other issues on the news agenda. Second, changes in the course of the pandemic—such as the introduction of vaccines— affect media attention. Third, this article illustrates how news media organisations try to balance informing the public of the risks related to the pandemic and not overreacting to the threat it poses.

## Introduction

This article explores the attention dynamics of the COVID-19 pandemic and illustrates the factors that cause a decline in the attention that pandemics receive. The article builds on 18 semi-structured interviews with Finnish journalists and on the literature on issue attention (Brosius and Kepplinger, 1995; Djerf-Pierre, 2012a, 2012b; Downs, 1972; Schäfer et al., 2014; Shih et al., 2008; Vasterman, 2005).

COVID-19 is a pandemic caused by the SARS-CoV-2 virus. The World Health Organization characterised COVID-19 as a pandemic in March 2020 (World Health Organization, 2022). As a sudden, widespread and deadly pandemic with a vast societal impact, COVID-19 met many of the dominant news criteria (see Harcup and O’Neill, 2017). The news media have covered the pandemic from various perspectives, ranging from the health impacts of the virus to restrictions and other policy responses (Furlan, 2021; Wirz et al., 2021). Research suggests that COVID-19 has harnessed considerable amounts of media attention. The pandemic has fuelled an uptick in the consumption of television and online news (Broersma and Swart, 2022; Van Aelst et al., 2021; Vermeer et al., 2022) and has even led to “doom-scrolling” (Mannell and Meese, 2022)—excessive consumption of pandemic-related news.

Through media content analysis, scholars have analysed media attention cycles during pandemics and epidemics. The amount of news media attention tends to reflect new cases, the emergence of new scientific evidence or new policy responses (Fox, 2021; Jung Oh et al., 2012; Shih et al., 2008). Media attention is often highly event-driven; however, some research suggests that the amount of media attention given to an issue is frequently uncoupled from real-world events (Djerf-Pierre, 2012a; Hawkins, 2011: 61–62; Schäfer et al., 2014; Vasterman, 2005: 510). For example, in analysing the news media attention to various infectious diseases, Shih et al. (2008: 157) argue that “the decrease in media attention did not necessarily mean the resolution of the problems”. In other words, pandemics can lose their newsworthiness despite the fact that infections still occur (Hooker et al., 2012: 225; Pearman et al., 2021).

However, less is known about how journalism practice affects the media attention to pandemics. Indeed, much of the scholarly work on infectious diseases and news media attention builds on quantitative data sets (Arendt and Scherr, 2019; Fox, 2021; Jung Oh et al., 2012; Santos-Gonçalves and Napp, 2022; Shih et al., 2008; Wirz et al., 2021). Qualitative data can help to illuminate how journalistic decision-making affects issue attention dynamics. This article contributes to the literature on pandemics and issue attention dynamics by building on 18 semi-structured interviews with Finnish journalists who have extensively covered the COVID-19 pandemic. The article provides insights on how journalism practice and journalistic routines affect issue attention dynamics. Such a perspective complements quantitative content analysis when examining, for example, the decoupling of media attention from the number of Sars-CoV-2 cases (see Pearman et al., 2021).

In this article, issue attention refers to the amount of attention paid to an issue by the news media. The fluctuations in news media attention are often referred to in the literature as issue attention cycles in which attention peaks are followed by waning news media attention (Downs, 1972). Issue attention often takes the form of self-enforcing news waves or news hypes in the news media (Djerf-Pierre, 2012b; Kepplinger and Habermeier, 1995; Vasterman, 2005; Wien and Elmelund-Præstekær, 2009). In attention cycles, a surge in news media attention is often followed by “issue fatigue” (Djerf-Pierre, 2012a: 501; Gurr and Metag, 2021) as news angles and sources are exhausted and the public interest in the issue diminishes. At the same time, issues compete for media attention: newsworthy issues with more news value displace previously newsworthy issues in the media agenda (Brosius and Kepplinger, 1995).

The article explores the attention dynamics of the COVID-19 pandemic and asks what feeds a decline in the news media attention that a pandemic receives. Three factors are identified. First, this article shows how issue fatigue and issue competition work to render the pandemic less newsworthy. Second, developments in the course of the pandemic affect the attention it receives from the news media (see Hooker et al., 2012; Wirz et al., 2021). Third, the attention given to a pandemic is affected by the fact that news media organisations strive to strike a balance between informing the public of the risks of the pandemic and alleviating the associated fear (see Klemm et al., 2019; Van Antwerpen et al., 2022).

The rest of this article proceeds as follows. First, an overview of the literature on issue dynamics is presented. Then, there is a recap of how Finland has been affected by COVID-19 and how the Finnish news media have covered the pandemic. After the data and method are presented, the results of the analysis are discussed. Finally, the article addresses the wider implications of this study for the scholarship on issue attention, discusses the limitations of this research and presents recommendations for future research.

## Issue attention dynamics

The amount of attention that an issue receives from the news media tends to fluctuate (Djerf-Pierre, 2012b; Downs, 1972; Jung Oh et al., 2012; Schäfer et al., 2014; Shih et al., 2008). Events that meet the dominant news criteria, such as negativity or wide societal relevance (Harcup and O’Neill, 2017), often trigger a “spiral of attention” (Djerf-Pierre, 2012a: 499), leading to increased levels of media coverage (Wien and Elmelund-Præstekær, 2009). In these circumstances, newsrooms direct their resources to the issue to produce follow-up stories on it and cover it from various perspectives (Vasterman, 2005). The cycle is strengthened by the fact that competing news media organisations pick up the issue, reinforcing its newsworthiness (Kitzinger and Reilly, 1997).

Research has also analysed the factors that contribute to the downward trend of issue attention cycles. After heightened levels of attention, issue fatigue gradually starts to settle in (Djerf-Pierre, 2012a: 501; Vasterman, 2005: 515). Issue fatigue refers to a development where news angles and sources are exhausted, and journalists and the public start to lose interest in the issue. In such cases, journalists might start experiencing difficulties in pitching stories to their editors (Djerf-Pierre, 2012a: 502). On the level of individual news consumers, a prolonged period of coverage leads to fatigue and declining interest (Djerf-Pierre, 2012a: 501; Kormelink and Gunnewick, 2022; Gurr and Metag, 2021: 1790). Combined, these factors contribute to a downward attention cycle.

Ultimately, news media attention is a zero-sum game. Newsroom resources are scarce, and the ability of the public to pay attention to issues is inherently limited (Brosius and Kepplinger, 1995; Rauchfleisch et al., 2021). Therefore, issues compete for attention in the news media (Brosius and Kepplinger, 1995; Geiß, 2011). Issue competition refers to the ability of more newsworthy issues to “crowd out” (Djerf-Pierre, 2012a) other issues from the news media agenda. For example, it has been argued that wars and economic crises can crowd out environmental issues from the news agenda (Djerf-Pierre, 2012a). Similarly, COVID-19 is a “killer-issue” (Geiß, 2011) that has had a negative impact on the news media attention that climate change receives (Rauchfleisch et al., 2021).

Scholars have specifically analysed the attention dynamics of epidemics and pandemics. It has been argued that the news media attention to these outbreaks is highly event-driven, meaning that new cases or governmental policy responses lead to spikes in news media attention (Arendt and Scherr, 2019; Jung Oh et al., 2012; Shih et al., 2008). A decrease in attention seems to be driven by the course of the disease and by the fact that issues must compete for scarce attention resources (Jung Oh et al., 2012: 227). Importantly, newsworthiness also declines when diseases turn out to be less harmful than initial expectations. Previous research shows how an avian influenza with pandemic potential eventually lost its newsworthiness as journalists deemed the influenza to be a “false alarm” (Hooker et al., 2012: 226).

Some research has already been done on the issue dynamics of the COVID-19 pandemic. While Fox (2021) finds differences in the COVID-19 coverage of Chinese, Korean, Taiwanese and Hong Kong newspapers, there are similarities as well. The amount of media attention reflected the geographic proximity of the virus, and media coverage declined as the spread of the virus abated (Fox, 2021). Analysing COVID-19 news coverage in U.S. and Chinese media, Wirz et al. (2021) find that the Chinese media coverage followed a standard issue attention cycle where attention peaks were followed by a decline in news media attention, whereas in the U.S., news media attention remained consistently high during the first months of the pandemic. Wirz et al. (2021: 15) thus suggest that when it comes to high impact pandemics such as COVID-19, traditional issue attention cycles might break down. This is supported by the notion that the attention the pandemic received in the Spanish media remained high during 2020 and 2021 (Santos-Gonçalves and Napp, 2022). However, some evidence suggests that after the initial peak of global media attention to COVID-19 in early 2020, the media coverage gradually waned and decoupled from the ongoing rise in the number of infections (Pearman et al., 2021).

Rauchfleisch et al. (2021) argue that the outbreak of COVID-19 in early 2020 crowded out climate issues from the news media. The pandemic arguably fit well with established news values; COVID-19 was a surprising, dangerous event that affected various aspects of social life, generating news stories from multiple perspectives (see Harcup and O’Neill, 2017: 1482). Attention was given not only to the number of cases, deaths and hospitalisation but also to the financial impacts of the pandemic, governmental pandemic responses and the shutdown of public life (Furlan, 2021: 127). The COVID-19 issue attention cycle has also been analysed from the perspective of news consumers. Based on interviews with Dutch youths, Kormelink and Gunnewick (2022) explain that increased consumption of COVID-19 related news was gradually replaced by “corona fatigue” and a return to standard levels of news consumption.

Through interviews with Finnish journalists who have closely followed the pandemic, this article further contributes to the literature on issue attention dynamics and COVID-19. First, however, it is necessary to recap the unfolding of the COVID-19 pandemic in Finland and how the pandemic has been covered by the Finnish news media.

## The Covid-19 pandemic in Finland and in the finnish news media

**Figure 1.**
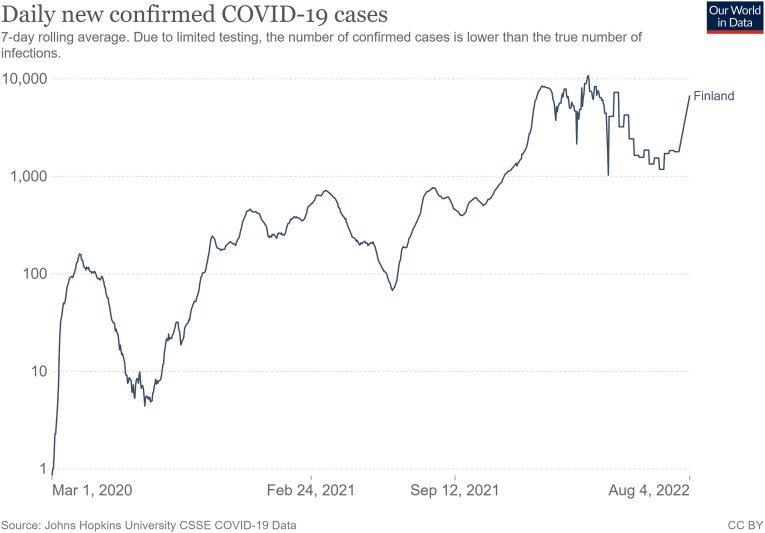
Daily new confirmed COVID-19 cases in Finland (logarithmic scale). Source: Our world in data. Retrieved from: https://ourworldindata.org/coronavirus/country/finland

In Finland, the first case of the SARS-CoV-2 virus was confirmed in late January 2020 when a Chinese tourist tested positive for the virus in Lapland, Northern Finland. In March 2020, the Finnish government introduced the Emergency Powers Act (Parliament of Finland, 2022). In spring 2020, various restrictions—ordered by the government as well as different regional and local authorities—were introduced to curb the spread of the virus. Summer 2020 saw many restrictions being lifted as the first wave of the pandemic receded (Figure 1). In autumn 2020, the number of cases started to climb again. In early 2021, the authorities had to resort to restrictions. The Emergency Powers Act was reintroduced in March 2021 and repealed in late April 2021.

In Finland, inoculations against the virus started in late 2020, and the rollout gathered momentum in 2021. Thus, authorities were able to lift restrictions despite the soaring number of infections driven by a fast-spreading variant of the virus. Spring 2022 finally saw the elimination of all remaining restrictions and recommendations.

News media has played a central role in providing people with relevant information regarding the pandemic. In Finland, as in many other European countries (Van Aelst et al., 2021), the pandemic led to a significant uptick in media consumption. Kantar, a market research company, reported that in spring 2020, the amount of time per day that Finns spent consuming media content rose by one and a half hours compared to the previous year (Kantar, 2020). The most popular sources of COVID-19 news have been TV news channels as well as the websites and mobile applications of Finnish mainstream news media (Matikainen et al., 2020: 86).

This article analyses the news media attention to the pandemic. In this respect, Finland is an interesting case study. The Finnish journalistic culture, often dubbed as a “democratic corporatist” culture (Hallin and Mancini, 2004), embodies many central tenets of professional Western journalism, such as a strong commitment to neutrality and professional self-regulation (Deuze, 2005a; Hallin and Mancini, 2004; Väliverronen, 2022). Moreover, various Finnish news media organisations have publicly reflected on how the pandemic should be covered. In September 2021, the most influential daily newspaper *Helsingin Sanomat* made the deliberate decision to put less emphasis on daily infection numbers in their COVID-19 coverage and rather focus on vaccination rates, deaths and hospitalisations. In an article reflecting on the reasoning behind the editorial shift, Esa Mäkinen, one of the managing editors of the paper, justified the decision by pointing to the increasing vaccination rates (Mäkinen, 2021). Mäkinen stated that COVID-19 news coverage should reflect a time when people “live with the virus”. Other news organisations, such as the public broadcaster *Yle* and the biggest private commercial television station *MTV*, made similar decisions (Ahtiainen, 2021; Luoma, 2021).

## Data and method

This article builds on 18 interviews with Finnish journalists who have extensively covered the pandemic. The semi-structured interviews were conducted in April and May 2022. The data-gathering process began when top journalists (for example, editors-in-chief and managing editors) from leading Finnish news organisations were contacted (Newman et al., 2021: 76–77). Those who agreed to be interviewed were asked to name colleagues who had closely followed the COVID-19 pandemic. The final data set consists of 18 interviewees, including journalists from the following news organisations: the biggest daily paper *Helsingin Sanomat,* the public broadcaster *Yle,* the most popular commercial TV channel *MTV* as well as the two Finnish tabloids *Iltalehti* and *Ilta-Sanomat*. Also included is *Lännen media*, a joint news organisation that produces news for various Finnish regional newspapers. The job descriptions and institutional positions of the interviewees vary from leading journalists (for example, editors-in-chief, managing editors and news editors) to reporters who focus on specific areas, such as domestic news, lifestyle or science.

The interviews were semi-structured and touched on various themes. For example, the journalists were asked to reflect on news work during the pandemic, communication practices of the Finnish government and the role of audience analytics (Lamot and Paulussen, 2020) in pandemic news coverage. Importantly, the journalists were asked to reflect on the fluctuations in news media attention.

The interviews took place both in person and online. The average length of the interviews was 50 min, with the shortest interview being 22 min and the longest 63 min. The interviews were recorded. The transcriptions were coded using the Atlas.ti qualitative analysis software. In the analysis, a thematic analysis approach was used (Mäenpää, 2014: 94–95). First, the interview material was coded. The open coding was done inductively with the theoretical framework in mind. The codes were then used to identify themes related to the media attention to the COVID-19 pandemic. To ensure interviewee anonymity, the interviewees are referred to using random numbers. Moreover, some of the direct quotations were slightly edited to ensure anonymity.

## Findings

This findings chapter is organised around themes that were identified from the material. The first concerns the interviewed journalists’ perceptions of the beginning of the pandemic and how COVID-19 became the overriding news theme across news desks and editorial departments (see Vasterman, 2005: 514). Subsequent themes illustrate the elements that caused a decline in the attention paid to the pandemic: issue fatigue and issue competition, developments in the course of the pandemic and editorial decisions regarding the scope and volume of pandemic coverage.

### From a pre-problem stage to an all-encompassing news theme

When asked about how their respective newsrooms started to realise the seriousness of the COVID-19 pandemic, many interviewees described what could be called, in Downs’ (1972: 39) terms, a “pre-problem stage” of the attention cycle where a “highly undesirable social condition exists” but has not yet captured much public attention. The interviewed journalists recalled how they received information regarding a novel virus in China in late 2019. They explained how, in the early stages of the attention cycle, the build-up towards rising levels of attention was driven by cascading events (Shih et al., 2008). New cases started to emerge in Europe, and the first cases of SARS-CoV-2 were detected in Finland in January 2020. The statement below shows the importance of the geographic proximity of the virus in garnering media attention (Fox, 2021: 1869):

*It was late 2019… I still remember it. We had had a story about a ‘mysterious virus spreading in China’, and it had been read a lot. […] We asked THL [The Finnish Institute for Health and Welfare] how concerned we should be. […]. Later, when there started to be infections in the Nordic countries… it was not a panic, but we started to wake up. […] When there was the first infection in Lapland, and we knew that it could spread fast, we were like, ‘Ok, who is going to Ivalo?’ [A county in Lapland, Northern Finland].* (Interviewee 15)

Another interviewee from a tabloid newspaper similarly tied the emergence of the first domestic cases to news organisations starting to prepare themselves for intensive pandemic coverage:

*It was the end of January, early February, because of the first case in Finland. Everyone was waiting for the second case, and it came pretty fast as well. Then we started to ‘oil the machine’, so to speak.* (Interviewee 14)

In the early stages of the pandemic, the media attention was fuelled by new cases and policy responses, echoing results of previous research on the media attention to infectious diseases (see Fox, 2021; Jung Oh et al., 2012; Shih et al., 2008). On 16 March 2020, the government declared a state of emergency. Schools were closed on 18 March 2020. One journalist pointed to these policy responses as a turning point in the media coverage.

*Finland was completely closed. Just like that. And that completely changed the tone of the stories as well as the volume.* (Interviewee 4)

The interview data supported the notion that in the early stages of an exceptional news event, newsrooms devote much of their resources to a single issue (Vasterman, 2005). Indeed, some of the interviewees revealed that “corona task forces” were established in their newsrooms, with the aim of covering COVID-19 from various journalistic perspectives. The pandemic became an all-encompassing theme across all news desks. One managing editor said that “50% of our content production has been about the pandemic” (Interviewee 18). An editor-in-chief claimed that the “psyche” (Interviewee 1) of a newsroom is built for situations where a single dominant news story is intensively covered from various perspectives. When asked about whether the newsroom was organised around COVID-19, a news editor confirmed that the whole team was covering the pandemic:

*Yeah, we had journalists concentrating on COVID-19 news. We had journalists covering the political side of things, domestic journalists, digital journalists, live coverage, journalists specialising in health issues and some foreign news reporters as well. But pretty quickly everyone was doing it. If you were a lifestyle journalist, you took topics that dealt with health or food or symptoms.* (Interviewee 2)

In many interviews, the COVID-19 pandemic was compared to a war. One editor-in-chief compared the COVID-19 crisis to the war in Ukraine launched by Russia in February 2022. Like the war, the pandemic was an “exceptional event” that demanded organisational adjustments in the newsroom.

*It was such an acute and exceptional event, and we understood—as with the war in Ukraine—that we must organise and build functions around it.* (Interviewee 5)

Research has shown that when more attention is paid to an issue, it tends to generate more interest (Djerf-Pierre, 2012b). Heightened levels of audience attention work to signal to the newsroom that the stories are worth doing. The interviews supported such dynamics. However, some interviewees downplayed the importance of audience analytics (Lamot and Paulussen, 2020) as a driver of COVID-19 coverage. One managing editor said that the role of audience analytics was rather minor in determining COVID-19 coverage. The interviewee said that it was evident that their blog-styled live article on the pandemic was very popular and thus worth doing. However, there was no need for nuanced audience data analysis.

*In the end, it’s* [the role of audience analytics] *pretty minor on such occasions. When we see that our daily numbers are two-, three- or even five-fold compared to normal traffic, those are big numbers. Everyone understands why those numbers are so big. You do not need anything too special. We just had to keep doing our work and put out good stuff. There was nothing special about that.* (Interviewee 18)

One journalist, in charge of a COVID-19 task force, said that during the pandemic, it was no longer necessary to use Chartbeat, an engagement analytics software widely used in newsrooms (Lamot and Paulussen, 2020):

*It* [Chartbeat] *shows you the amount of people reading a story simultaneously. And it is always looked at when someone publishes a story, and headlines are tweaked. However, during COVID-19, I gave up using it. I might have looked at it a couple of times, but the journalistic gut feeling was more important.* (Interviewee 9)

Others, however, referred to audience numbers as a driving force of COVID-19 media coverage (see Neheli, 2018; Tandoc Jr. and Thomas, 2015). One tabloid newspaper journalist said that when it came to working on the regularly updated COVID-19 live article, she was told “that it is your priority to keep this as our most read story” (Interviewee 15). Another interviewee from a tabloid newspaper said that reader statistics helped the newspaper scan for interesting stories and topics. For example, the first stories on the “beta” variant of the virus turned out to be widely read. This was a signal that more resources should be devoted to covering the emergence of new variants.

*The first news* [on the new variant] *received as many readers as the first wave of the pandemic, which told us that we needed to devote resources to this, because people are interested in everything about it. When one or two stories on an emerging issue tell us that people are interested, we start to think of follow-up stories for tomorrow and the day after that.* (Interviewee 12)

### Issue fatigue and issue competition

Reflecting on the shifts in news media attention, many interviewed journalists pointed to issue fatigue (Djerf-Pierre, 2012a; Kormelink and Gunnewiek, 2022; Gurr and Metag, 2021). After an intensive and long period of COVID-19 being covered from various perspectives, the journalists as well as the public gradually started to be worn out by the pandemic. One editor-in-chief described how after summer 2021, everyone was “damn tired” of the pandemic:

*We came back from the holidays last summer, and immediately we started to discuss how everyone was so damn tired of COVID-19 and whether there should be a change to scale down the coverage*. (Interviewee 1)

One news editor said that the coverage started to be “numbing” not only for the readers but for the journalists as well. COVID-19 left no room for any other ideas in the newsroom. According to the interviewee, it was important to remind “yourself” as well as the readers that there were other important things going on in the world as well.

*We felt that people needed other things as well. And how should the other things be weighted? That is about daily decisions. Should they be shown at the end of the* [TV news] *broadcast or at the bottom of the online news site? Or can we have things other than COVID-19 in the main news? That’s the discussion that we had.* (Interviewee 2)

The declining attention to COVID-19 was sometimes described as a natural phenomenon where the public interest in the pandemic gradually faded away. One journalist reminisced about pitching a story to a news editor about the first Finnish cases of the “omicron” variant of the virus in late 2021. “No one cares” was the response from the editor, but the story was among the most read stories for 5 days, the interviewee said. Another interviewee, who extensively covered the pandemic and was part of a COVID-19 task force, explained how the decline in attention was not driven by an editorial decision not to cover the pandemic but by a “natural” process:

*People stop caring or their attitude changes. They notice, through people they know, that the disease is not that serious after all. That’s how it goes. I think that is the best way; it fades away naturally from the media in accordance with the atmosphere. That’s better than the media making conscious decisions all the time that we will stop talking about this, because I do not think that is our role.* (Interviewee 11)

Similarly, a news editor from a tabloid newspaper said that public interest, reflected in readership numbers, drove coverage. When talking about the decision made by some Finnish news media outlets in autumn 2021 to scale down the attention paid to daily infection numbers, the interviewee stated that in their newsroom, the decision was “given to the reader”:

*[W]e had no reason to come out with a declaration that they* [the daily case numbers] *don’t matter. We gave the decision to the reader.* [*…*] *[W]e can see if the people are interested in COVID-19 restrictions, hospitalisations, intensive care case numbers or case numbers. That gives the decision-making power to the reader. We write about what is interesting, and gradually case numbers started to be less and less interesting.* (Interviewee 12)

However, it can be argued that editorial decisions regarding the scope and volume of COVID-19 coverage do not simply reflect reader preferences. Some interviewees said that the attention given to daily infections was scaled down despite the fact that the case numbers were followed closely by readers. Indeed, one managing editor said that the decision to stop routinely publishing daily case numbers was partly driven by the fact that they were so widely read. This points to the idea that the news media can alleviate fears associated with risks by adjusting their coverage and the amount of attention they pay to these risks (Bakir, 2010):

*There was still a considerable amount of interest in them* [the daily case numbers]*, but perhaps that was one of the reasons why we stopped* [publishing them]*.* (Interviewee 18)

According to the interviews, issue competition was also a major factor behind the declining levels of attention. Many interviewees explained how COVID-19 was “crowded out” (Djerf-Pierre, 2012a) by other news topics, most notably by the Russian attack on Ukraine in February 2022. One journalist said that the “Finnish COVID-19 discussion practically came to an end when Russia attacked Ukraine on 24 February” (Interviewee 13). Using similar phrasing, another interviewee stated that “in the media, the pandemic ended on 24 February 2022” (Interviewee 18). Another journalist simply stated that there was little interest in COVID-19 “now that we have Ukraine” (Interviewee 16). All available resources were devoted to covering the conflict:

*The war in Ukraine has taken all the resources from the domestic desk as well. It feels so stupid, but occasionally it feels like there can be only one main topic in the Finnish media. At times it is COVID-19, at times it is war. You cannot cover these simultaneously, which feels really stupid, but it is really just about resources.* (Interviewee 15)

Issue competition with the Ukraine war rendered the pandemic less newsworthy, despite the fact that COVID-19 related deaths were still frequent. One journalist reflected on why the latter did not receive much media attention from 2022:

*It’s just not on the news stream. There are mega issues around, and that’s probably one reason. But we have written about it* [COVID-19 deaths]. *We had a story on our front page a few days ago.* (Interviewee 3)

The gradual decline in interest in the pandemic was manifested in daily newsroom practices and discussions. A tabloid journalist described how the daily routine of covering infection numbers (see Kormelink and Gunnewiek, 2022: 679) went from a race—where newsrooms competed daily to be the first to publish the figures—to an almost irrelevant activity:

*Once, the news editors forgot to tell us whose responsibility it was to cover the COVID-19 numbers […]. Stuff like this happened, and we started to question what the point was.* [*…*] *Now, I think that we are supposed to have the numbers out on Thursdays, but I don't even know when was the last time we did it. That’s how it has been. Without any big declarations, it has just shifted.* (Interviewee 15)

### Developments in the course of the pandemic

According to the interviews, various developments in the pandemic, such as the introduction of vaccines and new mutations of the virus, also had an impact on media attention. Some interviewees stated that the emergence of the fast-spreading omicron variant of the coronavirus in late 2021 gradually rendered the number of infections less newsworthy. The fact that this variant appeared to be less deadly had an effect on media coverage (Hooker et al., 2012: 226). This supports the notion that from the perspective of newsrooms, the newsworthiness of the pandemic was gradually decoupled from the number of infections (see Pearman et al., 2021).

*With the omicron variant, the disease reportedly became less serious, causing fewer deaths and requiring less intensive care. We thought that the daily and weekly numbers were not as important anymore, although I felt that the readers would have liked us to keep reporting them.* (Interviewee 14)

Another interviewee stated how the invention and rollout of vaccines led to a situation where COVID-19 started to resemble the flu.

*[W]ithout the vaccinations, there was fear. That was in the beginning. But now you have vaccinations, and society works, and people do not wear masks anymore. It is not that different from a flu wave. You do not write about the flu.* (Interviewee 3)

However, the interviewees made it clear that the pandemic is likely to require attention from the news media in the future. For example, should new variants prove to be more severe or resistant to vaccines, the newsworthiness of the pandemic might increase. At the same time, it was argued that COVID-19 “has become a news topic among other topics”, as one managing editor put it.

*I think it will be covered* [in the future]*, but it has been normalised in many ways. Of course, should there be a new dangerous variant or a twist like that, or even a new pandemic… Sure, something like this will come in the future, but I think the premise is that should nothing extraordinary happen, it has become a news topic among other news topics.* (Interviewee 18)

### Alleviating fear

According to the interviews, the attention given to COVID-19 in the news media was also affected by news organisations’ attempts to balance different societal goals. In their respective newsrooms, leading journalists deliberated on the emphasis of pandemic coverage. One editor-in-chief talked about the attempt to balance the coverage of health risks and the need to “challenge” pandemic restrictions due to, among other things, the long-term mental health effects that they have on the youth:

*We talked a lot about our line* [regarding different aspects of the pandemic]. *In practice, our line is manifested by the emphasis of our news coverage. Regarding whether there is a need for stricter restrictions, we think about the stories that we are going to do and how many stories we are going to do* [*…*]. *Whether we are in favour of more or fewer restrictions is the end result of these stories.* (Interviewee 5)

In answering a question regarding pandemic coverage and shifting the emphasis away from daily COVID-19 numbers, one interviewee stated that the leading people in the newsroom “discussed it for weeks”. According to the interviewee, the problem was that a prolonged emphasis on daily infection numbers led to “alarmism” and fed “a culture of fear” in Finnish society.

*Maybe we tried to balance the societal mainstream that started to, how should I put this, overtly emphasise health security*. *Along the way, we tried to bring a wider societal perspective into the discussion. We also have things other than COVID-19 infections.* (Interviewee 8)

In a similar way, one managing editor said that questions regarding the editorial line on the pandemic were discussed “a lot” and on a “daily basis”. The interviewee stated that their aim was to be the “calm adult voice” in the discussion on COVID-19.

*For us, it was clear from the beginning that although the situation was severe, we should not overdo it. Protection from the disease is important, and there are other important issues in society as well.* [*…*]. *Of course, we talk about all kinds of opinions in the news.* [But] *a calm adult voice was probably the register that we aimed for during the whole crisis*. (Interviewee 18)

Retrospectively, some interviewees were critical of the decisions made in their organisations. One journalist argued that the decision to scale down reporting on the pandemic was driven by a willingness to “stop talking about COVID-19” (Interviewee 10). An editor-in-chief said that the decision in autumn 2021 to reduce COVID-19 coverage was driven by a justified attempt to increase plurality in a public sphere dominated by the pandemic. Later on, the newsroom had to ramp up coverage once again due to a new infection wave caused by the fast spread of the omicron variant. The interviewee was worried that the media aligned themselves with the Finnish government in wanting the pandemic to be over:

*In a way, it was wide-eyed hopefulness that we have gotten over this* [the pandemic]*.* (Interviewee 1)

## Conclusion and discussion

By building on the literature on issue attention dynamics (Djerf-Pierre, 2012a; Downs, 1972) and on 18 semi-structured interviews with Finnish journalists, this article analysed the issue attention dynamics of the COVID-19 pandemic and identified factors that cause the media attention to pandemics to decline. This article contributed to the issue attention literature by analysing how journalistic practices affect the fluctuations in the media attention to infectious diseases and the factors that contribute to a decline in media attention (Fox, 2021; Jung Oh et al., 2012; Shih et al., 2008).

The article started by illustrating how COVID-19 evolved to be an all-encompassing news theme in Finnish newsrooms in early spring 2020 and how news media organisations devoted much of their resources to pandemic coverage (Vasterman, 2005: 514). In this regard, this article supports the notion that the attention dynamics of COVID-19 have differed, to an extent, from other pandemics where peaks have been followed by sharp declines in attention. In contrast, COVID-19 has seen high levels of media attention over a longer period of time (Wirz et al., 2021). In Spain, for example, the interest of local journalists in the pandemic remained high throughout 2020 and 2021 (Santos-Gonçalves and Napp, 2022). This article also supports research that has found the geographic proximity of the virus to be a major driver of media attention to the COVID-19 pandemic (Fox, 2021). Indeed, the interviewed journalists described how newsrooms started to organise themselves around COVID-19 as the virus was detected in other Nordic countries and eventually in Finland.

In identifying the factors behind declining levels of media attention, the article first analysed the effects of issue fatigue and issue competition (Brosius and Kepplinger, 1995; Geiß, 2011; Gurr and Metag, 2021). The interviewees pointed to the fatigue caused by intensive pandemic coverage and the need to offer readers something else. Moreover, many of the interviewees described how the pandemic was eventually “crowded out” (Djerf-Pierre, 2012a) from the news agenda by the Russian attack on Ukraine in February 2022.

Second, this article illustrated how developments in the pandemic affected news media attention. The introduction and rollout of vaccines gradually rendered the pandemic less pressing as a news topic. The number of daily infections became less newsworthy, and, as one interviewee put it, the COVID-19 pandemic started to resemble the flu.

Third, this article identified how the attention devoted to the COVID-19 pandemic was affected by editorial decisions regarding the emphasis of COVID-19 coverage. In newsrooms, leading journalists discussed the emphasis of pandemic coverage and how the health risks posed by the virus should be covered compared to the long-term societal and economic effects of the pandemic and various restrictions. Indeed, this article argued that the media attention devoted to pandemics does not depend only on the course of the pandemic (Jung Oh et al., 2012; Pearman et al., 2021; Shih et al., 2008)); the findings indicate that at some point, news media organisations seek to alleviate some of the fears caused by the pandemic by steering the attention away from, for example, daily infection numbers. Such decisions should be further studied by scholars interested in the media attention to pandemics and other societal risks.

This study undoubtedly has certain shortcomings. Relying on interview material naturally runs the risk of sugar-coating the messy reality of daily news work. There might be a gap between journalists’ descriptions of attention dynamics and the actual amount of attention paid to pandemics. In the future, it would be interesting to analyse news media coverage of infectious diseases or other health risks by combining, for example, quantitative content analysis with ethnographic work among journalists. This would provide a better understanding of, for example, the effects of audience data analytics on coverage (see Lamot and Paulussen, 2020). In this regard, this article produced contradictory results. Some interviewees downplayed the importance of audience analytics where exceptional news events are concerned. However, others referred to audience numbers as a driving force of COVID-19 media coverage.

This article did not analyse the differences between various types of media organisations, partly to ensure interviewee anonymity. It should, however, be acknowledged that different kinds of news organisations are driven by somewhat differing journalistic cultures and economic incentives (Deuze, 2005b). Thus, the attention that a pandemic receives from a tabloid newspaper might differ from the coverage of a public broadcaster or prestigious newspaper.

Finally, it must be noted that the factors behind declining levels of media attention to pandemics are intertwined. For example, as indicated in the interviews, developments in the course of the pandemic—such as the rollout of vaccines and waning health risks—naturally fuel editorial decisions to scale down pandemic coverage. However, such developments do not necessarily dictate the attention that pandemics receive from the news media. This article suggests that a high death toll caused by a pandemic might garner low levels of news media attention should the pandemic have to compete with other attention-grabbing news issues. It is also possible that changes in the pandemic—the emergence of dangerous variants, for instance—might lead to a spiral of accelerating attention despite the attempts of news media organisations to avoid an atmosphere of fear. These dynamics should be studied in the future.
